# Beyond loot boxes: a variety of gambling-like practices in video games are linked to both problem gambling and disordered gaming

**DOI:** 10.7717/peerj.9466

**Published:** 2020-07-14

**Authors:** David Zendle

**Affiliations:** Computer Science, University of York, York, United Kingdom

**Keywords:** Problem gambling, Disordered gaming, Gaming disorder, Gambling-like practices in video games, Loot boxes, Esports gambling

## Abstract

A variety of practices have recently emerged which relate to both video games and gambling. These range from opening loot boxes, to esports betting, real-money video gaming, token wagering, and social casino spending. It is unknown either how harmful or how widespread many of these activities are. A sample of 1,081 adults from the UK aged 18+ was therefore recruited. This sample was purposively recruited via quota sampling to represent the UK population in terms of sex, age, and ethnicity. Engagement in all forms of gaming-related practices were significantly associated with both problem gambling and disordered gaming. A total of 18.5% of the sample had engaged in these activities at least once in the new year. These results suggest a convergent ecosystem of practices that relate to both video games and gambling. Engagement in each of these activities is linked to problem gambling. However, it remains unclear whether engagement in these activities causes problem gambling.

## Introduction

A blurring of the lines has occurred between video games and gambling activities. The most widely-discussed example of this convergence are loot boxes: Items in video games that may be bought for real-world money, but which contain randomized contents (Zendle et al., 2020).

Loot boxes share several formal features with gambling, and there has been widespread interest in the idea that engaging with loot boxes may lead to problem gambling (Brooks & Clark, 2019; Drummond & Sauer, 2018; King & Delfabbro, 2018; King & Delfabbro, 2020; Li, Mills & Nower, 2019). Indeed, engagement with loot boxes has been repeatedly linked to problem gambling: The more that gamers use loot boxes, the more severe their gambling problems tend to be (Brooks & Clark, 2019; Li, Mills & Nower, 2019; Zendle, 2019a; Zendle et al., 2018; Zendle, Meyer & Over, 2019; Zendle & Cairns, 2018; Zendle & Cairns, 2019). It is important to note that the robustness of this link does not necessarily mean that loot box spending causes problem gambling: It may well be the case that these factors are instead linked because problem gamblers are more likely to engage with loot boxes, or that some third factor (such as impulsivity) drives engagement with both loot boxes and gambling activities.

Problem gambling refers to a pattern of gambling engagement that is so extreme it causes an individual to have important problems in various aspects of their life (Raylu & Oei, 2004). It has been linked to depression, anxiety, bankruptcy, and suicidality (Barrault & Varescon, 2013; Grant et al., 2010; Petry & Kiluk, 2002). It is commonly considered a serious public health issue, and the similarities and associations between loot boxes and gambling have been sufficient to lead to global regulatory interest in loot boxes. For example, in the United Kingdom, similarities regarding loot boxes prompted a parliamentary inquiry into their effects (Kent, 2019). Evidence of their relationship to gambling was sufficient to prompt the UK government to announce a planned holistic reform of gambling law in order to address issues related to loot boxes(Hymas, 2019).

However, loot boxes are not alone in blurring lines between gambling and video games. A variety of gambling and gambling-like practices have recently emerged in the video game domain. These range from betting on esports, to spending money on social casino games, to more obscure practices like token wagering and real-money video gaming.

As noted above, there are a variety of different practices that constitute an intersection of gambling and video games. Given the interest that has been shown to the intersection of gambling and video games that loot boxes constitute, one might imagine that a rich literature exists to explore questions of prevalence and potential harm when it comes to these activities. However, little research has attempted to estimate either how common several of these practices are, or whether they share a link to problem gambling in a similar fashion to loot boxes.

Researchers have proposed that loot boxes share so many formal similarities with gambling that they may act as a gateway to engagement with gambling amongst gamers, and hence the development of problem gambling (Drummond & Sauer, 2018). Similarly, researchers have proposed that loot box spending may be linked to disordered gaming. Disordered gaming refers to a possible condition in which persistent and recurrent engagement with video games leads to significant impairment or distress (American Psychiatric Association, 2013). Such a relationship may lead to a problematic situation in which vulnerable individuals who play games to an excessive degree may be exposed to other potential harms (Li, Mills & Nower, 2019).

Similar concerns may be raised about other gambling and gambling-like practices that are associated with video games. An overview of each of these relevant practices is given below, as is an overview of research into engagement with loot boxes.

### Esports betting

Esports betting refers to the practice of placing wagers on the outcomes of multiplayer videogame competitions. Esports spectatorship has become increasingly popular in recent years. Indeed, the final of the 2018 *League of Legends* World Championships attracted over 44 million concurrent viewers (Goslin, 2018). This has led to a rapidly-growing betting culture, with mainstream betting providers commonly hosting esports streams alongside traditional sports like rugby and football. One industry report estimates that $5.5 billion was bet on esports in 2016 alone, with this figure set to more than double to $12.9 billion by 2020 (Smith, 2019).

Research has suggested that esports betting may be linked to esports spectatorship and video gaming, and that esports betting may be linked more strongly to problem gambling than traditional sports betting (Gainsbury, Abarbanel & Blaszczynski, 2017; Macey & Hamari, 2018). However, the overall prevalence of esports betting is unknown.

### Social casino games

In social casino games, players can spend real money to engage in simulated games of chance such as roulette or slot machine play. These games are commonly presented and sold in mobile phone app stores as a form of video game. They differ from conventional gambling in that players cannot win real-money rewards for their spending (Gainsbury et al., 2014). There is evidence that users of social casino games may migrate to engagement in conventional gambling activities (Gainsbury et al., 2016). Some social casino providers claim to have millions, or tens of millions, of active players (Kim et al., 2015). The prevalence of social casino spending may therefore be high. A recent large-scale survey of Canadian adolescents (*n* = 10,035) found that as many as 12.4% of respondents had recently played social casino games (Veselka et al., 2018). However, the generalisability of this figure to other populations is unclear.

### Real-money video gaming

Real-money video games integrate the ability for gamers to wager real money on the outcomes of their in-game efforts. For example, in *Strike! eSports Bowling*, players are prompted in-game to wager money on their success at games of online ten-pin bowling. Video games incorporating this mechanic range from sports games to Tetris-like puzzles such as *Block Blitz* (Zendle, 2019b). The prevalence of engagement in these activities and links to engagement with gambling are unclear.

### Token wagering

In games that incorporate token wagering, players do not wager real-world money on the outcome of their in-game activities. Instead, they engage in the related practice of wagering tokens or points. The rewards from winning wagers can then be redeemed for in-game rewards. This form of wagering is common in popular games such as *DOTA 2,* which is estimated to have over 11 million unique players each month (Valve Corporation, 2019; VPEsports, 2020). After conducting a literature search, we have failed to find a single academic source which examines either the potential effects or the prevalence of token wagering.

### Loot boxes

Loot boxes are items in video games that are bought with real-world money but contain randomised contents whose value is uncertain at the point of purchase. For example, players of the first-person shooter game *Counter-Strike: Global Offensive* can spend money to purchase sealed ‘weapon cases’. The contents of these cases may be rare and valuable, or they may be common and worthless. Players are not aware of the value of loot box contents when they make their purchase. Because of formal similarities between loot boxes and gambling, there are concerns that they may provide a gateway to gambling amongst gamers (Drummond & Sauer, 2018). Research has repeatedly linked loot box spending to problem gambling, with effect sizes in excess of *η*^2^ = 0.04 repeatedly observed in the literature (Brooks & Clark, 2019; Zendle, Meyer & Over, 2019; Zendle & Cairns, 2018; Zendle & Cairns, 2019). In media effects research, it is common to refer to effects in excess of this cut-off as potentially ‘clinically significant’ in nature, as effects smaller than this may be of insufficient magnitude to warrant a clinician’s attention (Ferguson, 2009). However, the temporal order of these links is unclear: They may represent a case in which loot boxes act as a gateway to problem gambling; alternatively, they may represent a case in which pre-existing gambling problems causes gamers to spend more money on loot boxes (Zendle, Meyer & Over, 2019). Indeed, they may represent a situation in which a third factor is responsible for both behaviours: For example, those who have access to loot boxes may also tend to have access to similar technology-driven forms of gambling such as internet casinos and bingo.

One may also speculate that the financial cost associated with loot boxes may be so low as to not be potentially harmful. Indeed, recent surveys have shown relatively low mean spending on loot boxes amongst gamers (Drummond et al., 2020; Zendle & Cairns, 2018). However, the range of spending on loot boxes in video games is far from certain. The analyses highlighted above were conducted over samples of limited representativeness: Zendle and Cairns, for example, recruited participants from reddit, a popular online bulletin board. Using their results to estimate mean spend may be of limited utility.

Loot box spending has also been linked to disordered gaming, suggesting that it may be more common amongst vulnerable gamers (Li, Mills & Nower, 2019). No studies have thus far examined what proportion of the population engage in this behaviour. The prevalence of loot box spending is therefore unclear.

### Watching gambling and loot box spending online

Live game streaming services like *Twitch* and video sharing websites like *YouTube* allow individuals to broadcast both live and pre-recorded videos online. These platforms are also commonly used to broadcast live videos of individuals engaging in both gambling activities, and opening loot boxes (Kent, 2018; Klepek, 2019). The prevalence of watching such streams, and the links between this kind of engagement with both video game play and gambling is unclear.

### Summary

The lines between video games and gambling have been thoroughly blurred. A variety of practices exist that incorporate elements of both video games and gambling: esports betting, social casino spending, real-money gaming, token wagering, loot box spending, and the watching of gambling and loot box opening videos online. Excepting loot box spending, both the prevalence of these practices and their links with potentially important factors such as problem gambling and disordered gaming are currently unclear. The objective of this study is to begin investigating these issues.

In the present research, we therefore measure engagement in the variety of gambling-related video game practices outlined above. We then estimate both the prevalence of these practices; and we measure the relationship between each of these practices and both problem gambling and disordered gaming.

## Materials & Methods

### Design

We conducted an online survey with a sample of adults aged 18+ from the United Kingdom. This sample was recruited for us by Prolific Academic, and was quota-sampled to be nationally representative in terms of ethnic, sex, and age subgroups as calculated by the 2011 UK Census. Data were collected remotely via Prolific, and researchers did not have control over who was selected for inclusion within the sample, reducing the potential for selection bias.

Prolific Academic are an online panel provider. The participant pool for Prolific Academic consists of a group of individuals who have previously signed up with this service provider in order to take part in experiments and other studies. These participants may check their Prolific accounts at any point that is convenient to them. These accounts will then list studies that are available for them to take part in. After participation, individuals are remunerated for their efforts. In cases such as this, recruitment takes the form of offering relevant individuals (i.e., those that match the quota sampling frame described in this document) the opportunity to take part in this study.

Interestingly, this format means that traditional response rates cannot be obtained in this context. In a traditional survey (such as a ‘mail shot’), researchers might physically post out 1,000 envelopes to participants. If they received 900 responses from this initial invitation, they would be able to quantify a response rate of 90%. There is no easily-accessible analogue in the context of online providers like Prolific and Amazon Mechanical Turk. If, for example, an Amazon Mechanical Turk study is available to a million mTurkers and is filled in by a sample of 1,000, does this correspond to a participation rate of 0.1%? We would argue that it does not.

### Measurements

The frequency of participants engagement with gambling activities were measured by asking participants how frequently they had engaged in a variety of behaviours during the past 12 months. Answers to these questions were measured on an 8-point scale: (1) I have never done this; (2) Not at all in the past 12 months, but I have done this before then; (3) Less than 10 times in total; (4) Once a month; (5) 2–3 times a month; (6) Once a week; (7) 2–3 times a week; (8) 4 or more times a week;. 11 traditional forms of gambling were measured. These were:

1. Purchasing lottery tickets
2. Purchasing instant win/scratch cards
3. Betting on sports events (excluding esports)
4. Betting on horse or dog racing
5. Playing bingo for money in person
6. Playing bingo for money online
7. Playing games of skill for money against other individuals
8. Playing slot machines in person
9. Playing slot machines online
10. Playing casino table games in a casino
11. Playing casino table games online

In addition to this, gaming-related forms of gambling and gambling-like behaviour were measured via the same procedure. The forms of gaming-related behaviour that were measured were:

1. Esports betting (“Esports betting is the practice of wagering real money on the outcome of video game competitions or matches. Over the past 12 months, how often would you say that you have engaged in esports betting?”)
2. Loot box spending (Question given below)
3. Social casino spending (“Social casino games refer to apps in which players can play simulated gambling games. Examples of such games are Zynga Poker and Pop! Slots. These games differ from traditional gambling because whilst players may pay real-world money to play these games, they cannot receive monetary rewards from them. Over the past 12 months, how often would you say that you have spent money in social casino games?”)
4. Real-money video gaming (“Real-money video games are video games where you can wager real money on your in-game success, in the hopes of winning more real money. Examples of such games include Strike! Bowling, and Pro Pool. Over the past 12 months, how often would you say that you have spent money on real-money video games?”)
5. Token wagering (“Token wagering is a term that is used to refer to the practice of wagering tokens or points on the outcome of video game competitions or matches. Over the past 12 months, how often would you say that you have engaged in token wagering?”)
6. Watching loot box openings online (live)
7. Watching loot box openings (pre-recorded)
8. Watching gambling online (live) (“Some people watch others gamble live via video streaming service or websites like Twitch. Over the past 12 months, how often would you say that you have engaged in watching others gamble live via a video streaming service or website?”)
9. Watching gambling online (pre-recorded) (“”Some people watch others gamble via pre-recorded clips on websites like YouTube. Over the past 12 months, how often would you say that you have engaged in watching others gamble via a pre-recorded clip on a website?)

In order to measure loot box spending, a more time-consuming procedure was employed. Participants were first given the following description of loot boxes:

“’Loot boxes’ refer to any items or rewards in a video game that are paid for with real money, but contain randomised contents. Not all loot boxes literally look like boxes. For example:

• Players of Counter-Strike: Global Offensive can pay real-world money to open ’weapon cases’ that contain a random skin for their in-game gun. Some skins are more rare than others.
• Players of FIFA Ultimate Team can pay real-world money to buy ’player packs’ of new footballers. The contents of player packs are randomised, and when paying their money, gamers don’t know if they’re paying for good players or poor players.
• Players of Fire Emblem: Heroes can pay real-world money for the chance to obtain a random in-game hero.“

They were then asked “Given the definition of loot boxes above, have you played a game during the last 12 months where you had the opportunity to buy a loot box?” and “Over the past 12 months, how often would you say that you have purchased loot boxes?”

It is important to note that in order to ensure that participants gave accurate responses, each of these novel forms of gaming-related behaviour was introduced to participants via a short piece of text prior to asking participants to endorse an answer. For example, esports betting was introduced with the text “Esports betting is the practice of wagering real money on the outcome of video game competitions or matches.”; watching gambling online (live) was introduced with the text “Some people watch others gamble live via video streaming service or websites like Twitch”. The exact questions asked for each item surveyed are available at the OSF repository listed in the data accessibility statement for this manuscript.

Two aggregate measures of both **engagement in any traditional form of gambling** and **engagement in any form of video game and gambling-related activity** were formed by taking a participant’s maximum response to either any of the traditional gambling frequency measures, or any of the video game-related frequency measures. In order to be as parsimonious as possible with estimating the frequency of video game-related gambling and gambling-like behaviour, both watching gambling live and on a pre-recorded basis were excluded from this calculation.

**Problem gambling severity** was measured through administration of the problem gambling severity index (PGSI). This 9-item scale is commonly used to measure problem gambling, and has been extensively validated (Ferris & Wynne, 2001; Orford et al., 2010; Turner et al., 2018). Each question in the PGSI measures how frequently an individual has engaged in a problematic gambling-related behaviour during the past year (e.g., “Thinking about the last 12 months, how often have you bet more than you could really afford to lose?”). Answers are given on a four-point scale ranging from “Never” to “Almost always”, and scores are summed to create an overall index of problem gambling severity ranging from 0 to 27. In this instance, diagnostic categories are formed according to (Currie, Hodgins & Casey, 2013): Individuals who score 0 on the PGSI are categorised as ‘non problem gamblers’; 1–4 as ‘low risk gamblers’; 5–7 as ‘moderate risk gamblers’ and 8+ as ‘problem gamblers’. Other scoring schemes for the PGSI calculate low risk gamblers and moderate gamblers differently; there is some evidence that this scheme yields greater validity (Currie, Hodgins & Casey, 2013). The Cronbach’s Alpha for this scale was calculated in this instance as 0.85.

**Disordered gaming** was measured via administration of the Internet gaming disorder scale (Lemmens, Valkenburg & Gentile, 2015). This 9-item instrument presents a series of Yes/No questions regarding the activities that individuals have engaged in during the past year that map to APA (American Psychiatric Association) criteria for the presence of gaming disorder (e.g., “Have you had arguments with others about the consequences of your gaming behaviour?”). Endorsement of 5 or more criteria is used to screen individuals as positive for the presence of disordered gaming. This is based on diagnostic criteria for Internet Gaming Disorder found in the 5th edition of the Diagnostic and Statistical Manual for Mental Disorders. The KR-20 for this scale was calculated in this instance as 0.79.

Ethical approval for this research was granted by the York St John University Cross-Departmental Ethics Board, under submission code 2,159. It should be noted that the lead author’s current affiliation is to the University of York, and not York St. John University. The reason for the difference in the IRB and the current author’s host institution is that this study was conducted during the author’s transition from a lectureship at York St. John University to a lectureship at the University of York.

Informed consent was gathered from each participant. As this was an online study, this took the form of a brief description of the kinds of data that would be collected and how it would be used, and an opportunity to opt in to the study by ticking a box. The full script for this study (including the specific presentation of this informed consent procedure) is available at the OSF repository detailed below.

### Participants

Overall, an initial sample of 1,201 individuals was collected between August 30th 2019 and September 9th 2019. Participants were recruited via a deliberately ambiguous study descriptor that was designed to minimize any potential for self-selection bias. This message read as follows: “In this study you will be asked to provide some demographic details, and then some information about activities that you engage in.”. In addition to the 1,201 participants whose data was collected, a further 21 individuals began, but then returned the study.

Participants were reimbursed £0.70 for taking part in the study. On average, participants took less than 8 min to complete and submit their responses to the study, with an average reimbursement rate of £5.65/hr.

After data were recorded, participants were then excluded from the sample on the basis of either (a) failing an initial seriousness check or (b) failing either of two later attention checks.

First, participants were excluded on the basis of their response to a seriousness check. This took the form of a series of four questions asking participants whether they had played specific games. Two games listed (‘Game of Glory’ and ‘Rise of Warriors’) were fictitious. Any participants who indicated that they had played these games were removed from the sample. Thirty–nine participants endorsed these items and were removed from the sample.

The attention check took the form of two separate questions which asked participants to give pre-specified answers in order to establish that they were paying attention to the survey. The first attention check question read “In order to check the reliability of your responses, please select ‘Once a month’ as the answer to this question”. The second attention check question read “Please select ‘No’ as the answer to this question”. Eighty participants failed the first attention check, and a further one participant failed the second. These participants were removed from the study, leaving a total of 1,081 participants.

Overall, 526 participants listed their gender as male, and 549 listed their gender as female. Six gave other responses.

Overall, 926 participants listed their ethnicity as White; 29 as Mixed; 38 as Black; 76 as Asian, and 12 as Other.

Overall, 190 participants were aged 18–27, 176 participants were aged 28–37, 203 participants were aged 38–47, 184 participants were aged 48–57, and 328 participants were aged 58+.

Overall, 24 of the 1,081 participants (2.2%) were classified as problem gamblers. A further 35 (3.2%) were classified as moderate risk and 235 (21.7%) as low risk. Seventy–nine of the 1,081 participants (7.3%) were classified as disordered gamers.

Overall, 13 of the 526 male participants (2.4%) were classified as problem gamblers, and 11 of the 549 female participants (2.0%). None of the six participants giving other responses when asked about their gender were problem gamblers. Forty–nine of the 526 male participants (9.3%) were classified as disordered gamers, and 30 of the 549 female participants (5.4%). None of the participants who gave other gender responses were classified as disordered gamers.

Seventy–two individuals had both engaged in traditional gambling and loot box spending in the past year. The majority of these (42) stated that they had engaged in traditional gambling prior to loot box spending; 17 had engaged in loot box spending prior to traditional gambling; and 13 responded ‘Not sure/Not applicable’ when asked.

Thirty–five individuals had both engaged in traditional gambling and social casino spending in the past year. The majority of these (23) stated that they had engaged in traditional gambling prior to social casino spending; four had engaged in social casino spending prior to traditional gambling; and eight responded ‘Not sure/Not applicable’ when asked.

Thirty–one individuals had both engaged in traditional gambling and esports gambling in the past year. The majority of these (23) stated that they had engaged in traditional gambling prior to esports gambling; four had engaged in esports gambling prior to traditional gambling; and four responded ‘Not sure/not applicable’ when asked.

Twenty individuals had both engaged in traditional gambling and token wagering in the past year. The majority of these (12) stated that they had engaged in traditional gambling prior to token wagering; two had engaged in token wagering prior to traditional gambling; and six responded ‘Not sure/ Not applicable’ when asked.

## Results

The proportion of the sample engaging in each of the traditional gambling and gaming-related gambling behaviours within the sample is shown below as Table 1. 95% confidence intervals were calculated via the adjusted Wald procedure.

**Table 1:** Prevalence estimates for both traditional gambling and gaming-related gambling behaviours, with 95% confidence interval calculated according to the adjusted Wald procedure.

**Type of behaviour**	**Behaviour**	**Proportion engaging in activity at least once in the past 12 months**	**LLCI**	**ULCI**
Gambling-like video game practices	Any form of gambling-like video game practice	18.5%	16.3%	20.9%
	Esports betting	2.9%	2.0%	4.1%
	Loot box spending	7.8%	6.4%	9.6%
	Social casino spending	3.7%	2.7%	5.0%
	Real-money video gaming	1.7%	1.1%	2.7%
	Token wagering	2.2%	1.4%	3.3%
	Watching loot box openings online (live)	4.8%	3.6%	6.2%
	Watching loot box openings online (pre-recorded)	6.6%	5.3%	8.3%
	Watching gambling online (live)	4.1%	3.1%	5.5%
	Watching gambling online (pre-recorded)	4.2%	3.1%	5.6%
Traditional forms of gambling	Any form of traditional gambling	71.3%	68.5%	73.9%
	Purchasing lottery tickets	48.9%	45.9%	51.9%
	Purchasing instant win / scratch cards	34.7%	32.0%	37.6%
	Betting on sports events (excluding esports)	27.6%	25.0%	30.4%
	Betting on horse or dog racing	21.7%	19.3%	24.3%
	Playing bingo for money in person	8.2%	6.7%	10.0%
	Playing bingo for money online	11.1%	9.4%	13.2%
	Playing games of skill for money against other individuals	6.8%	5.4%	8.5%
	Playing slot machines in person	13.3%	11.4%	15.4%
	Playing slot machines online	14.4%	12.4%	16.6%
	Playing casino table games in a casino	7.4%	5.9%	9.1%
	Playing casino table games online	10.4%	8.7%	12.4%

Spearman rank correlation coefficients were calculated for the relationship between ordinal responses (1–8) to questions regarding the frequency of engagement with each form of game-related practice and both problem gambling and disordered gaming. The result of this analysis is presented below as Table 2. In all relevant instances, continuous scores were calculated from the IGDS by counting the presence of a symptom as ‘1’ and the absence of a symptom as ‘0’, forming a numeric score ranging from 0 to 9.

**Table 2:** The relationship of gambling-like video game practices with both disordered gaming and problem gambling.

**Gambling-like video game practice**	**Relationship with problem gambling (Spearman’s rho)**	**Relationship with disordered gaming (Spearman’s rho)**
Any form of gambling-like video game practice	0.23^***^	0.43^***^
Esports betting	0.21^***^	0.14^***^
Loot box spending	0.14^***^	0.41^***^
Social casino spending	0.21^***^	0.17^***^
Real-money video gaming	0.15^***^	0.18^***^
Token wagering	0.12^***^	0.18^***^
Watching loot box openings online (live)	0.14^***^	0.32^***^
Watching loot box openings online (pre-recorded)	0.13^***^	0.35^***^
Watching gambling online (live)	0.20^***^	0.30^***^
Watching gambling online (pre-recorded)	0.17^***^	0.25^***^

**Notes.**

* Relationships that are significant at the *p* < 0.05 level are marked.

** *p* < 0.01 are marked.

*** *p* < 0.001 are marked.

Spearman rank correlation coefficients were calculated for the relationship between ordinal responses (1–8) to questions regarding the frequency of engagement with each traditional form of gambling and each form of game-related practice. The results of this are given below as Table 3.

**Table 3:** Spearman’s rho correlation coefficient between engagement in traditional gambling activities and engagement in gaming-related gambling activities.

	Esports betting	Loot box spending	Social casino spending	Real-money video gaming	Token wagering	Watching loot box openings online (live)	Watching loot box openings online (pre-recorded)	Watching gambling online (live)	Watching gambling online (pre-recorded)
Any form of traditional gambling	0.09^**^	0.04	0.13^***^	0.07^*^	0.03	<0.01	<0.01	0.03	0.07
Purchasing lottery tickets	−0.02	−0.05	0.07^*^	0.05	−0.03	−0.08^**^	−0.09^**^	−0.04	−0.03
Purchasing instant win / scratch cards	0.08^**^	0.07^*^	0.15^***^	0.13^***^	0.06^*^	0.04	0.01	0.06^*^	0.05
Betting on sports events (excluding esports)	0.18^***^	0.08^**^	0.17^***^	0.12^***^	0.07^*^	0.03	0.02	0.05	0.08^**^
Betting on horse or dog racing	0.07^*^	0.01	0.09^**^	0.06^*^	0.04	−0.04	−0.07	0.05	0.02
Playing bingo in person for money	0.08^**^	0.03	0.12^***^	0.13^***^	0.06^*^	<0	0.01	0.07^*^	0.08^**^
Playing bingo online for money	0.14^***^	0.07^*^	0.19^***^	0.21^***^	0.08^**^	0.05	0.06^*^	0.09^**^	0.07^*^
Playing games of skill for money	0.22^***^	0.27^***^	0.22^***^	0.17^***^	0.16^***^	0.19^***^	0.18^***^	0.28^***^	0.24^***^
Playing slot machines in person	0.08^**^	0.12^***^	0.17^***^	0.11^***^	0.06^*^	0.05	0.07^*^	0.14^***^	0.12^***^
Playing slot machines online	0.23^***^	0.16^***^	0.26^***^	0.19^***^	0.09^**^	0.12^***^	0.13^***^	0.14^***^	0.12^***^
Playing casino table games in a casino	0.13^***^	0.13^***^	0.21^***^	0.09^**^	0.06^*^	0.05	0.04	0.12^***^	0.10^***^
Playing casino table games online	0.24^***^	0.21^***^	0.28^***^	0.22^***^	0.18^***^	0.12^***^	0.13^***^	0.20^***^	0.20^***^

**Notes.**

* Relationships that are significant at the *p* < 0.05 level are marked.

** *p* < 0.01 are marked.

*** *p* < 0.001 are marked.

Spearman rank correlation coefficients were calculated for the relationship between ordinal responses (1–8) to questions regarding the frequency of engagement with each video-game related gambling practice and each other game-related practice. The results of this are given below as Table 4.

**Table 4:** Spearman’s rho correlation coefficient between engagement in each gambling-like video game practice.

	Esports betting	Loot box spending	Social casino play	Real-money video gaming	Token wagering	Watching loot box openings online (live)	Watching loot box openings online (pre-recorded)
Esports betting	1	0.16^***^	0.28^***^	0.25^***^	0.31^***^	0.25^***^	0.21^***^
Loot box spending		1	0.2^***^	0.2^***^	0.24^***^	0.44^***^	0.42^***^
Social casino spending			1	0.38^***^	0.34^***^	0.25^***^	0.21^***^
Real-money video gaming				1	0.4^***^	0.23^***^	0.2^***^
Token wagering					1	0.35^***^	0.32^***^
Watching loot box openings online (live)						1	0.69^***^
Watching loot box openings online (pre-recorded)							1

**Notes.**

* Relationships that are significant at the *p* < 0.05 level are marked.

** *p* < 0.01 are marked.

*** *p* < 0.001 are marked.

### Exploratory analyses

During peer review, a number of additional analyses were suggested by reviewers. We agreed with these reviewers that these analyses constituted an interesting exploration of the data, and report them below.

To begin with, it was suggested that we scrutinize our data for evidence of common method bias. Common method bias occurs when shared variance amongst variables is attributable to a common method used to measure these items. Lengthy and demanding procedures are thought to lead to common method bias, as individuals attempt to optimize their progress through a study by using a method other than valid responding (MacKenzie & Podsakoff, 2012). The simplicity and short length of this study (less than 10 min mean completion time) may mitigate the risk of common method bias in our results. However, in order to address this issue, Harman’s Single Factor Test was applied to our data. This is a common test for common method bias (Podsakoff et al., 2003). It involves entering all measured variables into an exploratory factor analysis in order to determine how much variance between items is attributable to a single unifying factor. A measurement above 50% is taken as evidence that common method bias may be present. However, in this case, a single factor was only able to explain 23.44% of variance. The limitations of this approach are described in our discussion.

Next, reviewers requested that analyses investigating the relationship between the novel activities under investigation and both problem gambling and disordered gaming were conducted that statistically took age and gender into account. In order to binarise gender, we removed the 6 members of the cohort who identified as neither male nor female before analyzing the data.

In many studies that address the predictors of problem gambling, such an analysis would be conducted via multiple linear regression. However, inspection of a plot of the residuals of an initial analysis revealed that the strongly non-normal nature of our outcome variable meant that any models build on this data would violate assumptions of multivariate normality. In order to address this, data was analysed via logistic regression-based approaches, which do not require this assumption.

First, the ability of age, gender, and gaming-related behaviours to predict problem gambling were analysed via a series of ordinal logistic regressions. Each regression had problem gambling (non-problematic, low-risk, moderate-risk, problem gambler) as predictor. Each analysis had gender, age, and the frequency of a single form of gaming-related behavior as outcomes. Assumptions of multicollinearity were addressed via the calculation of VIFs for each regression; in each case, all VIFs were below 4, indicating a lack of multicollinearity. Assumptions of proportional odds were tested via the calculation of Brant’s test for each ordinal logistic regression: in all cases, omnibus test statistics were non-significant (*p* > 0.05), indicating a failure to reject the null hypothesis that the relationship between each pair of outcome groups is the same. All *p*-values for all predictors across all analyses were also non-significant, with the exception of token wagering (*p* = 0.02). For this reason, analyses involving token wagering are not reported below. Results of regressions involving problem gambling as outcome are depicted below as Table 5.

**Table 5:** Ordinal logistic regression of the influence of gaming-related behaviours, gender, and age on problem gambling. Odds Ratios are reported as a measure of effect size. These represent the influence of a single point difference in a factor on the likelihood that an individual is in a group other than non-problem gamblers.

Gaming-related behavior					Gender				Age			
Behaviour name	*β*	SE	t	OR	*β*	SE	t	OR	*β*	SE	t	OR
Esports betting	0.55	0.09	5.87^***^	1.73	−0.01	<0.01	−2.76^**^	0.99	0.41	0.14	2.92^**^	1.50
Loot box spending	0.31	0.09	3.37^***^	1.36	−0.01	<0.01	−2.60^**^	0.99	0.38	0.14	2.74^**^	1.47
Social casino play	0.62	0.10	6.31^***^	1.87	−0.01	<0.01	−2.78^**^	0.99	0.40	0.14	2.85^**^	1.49
Real-money video gaming	0.58	0.15	3.77^***^	1.79	−0.01	<0.01	−3.13^**^	0.99	0.42	0.14	3.01^**^	1.52
Token wagering	0.46	0.13	3.54^***^	1.59	−0.01	<0.01	−2.97^**^	0.99	0.41	0.14	2.98^**^	1.51
Watching loot boxes (live)	0.43	0.10	4.37^***^	1.54	−0.01	<0.01	−2.32^*^	0.99	0.37	0.14	2.61^**^	1.44
Watching loot boxes (pre-recorded)	0.39	0.09	4.14^***^	1.48	−0.01	<0.01	−2.02^*^	0.99	0.36	0.14	2.56^*^	1.43
Watching gambling online (live)	0.47	0.09	5.24^***^	1.60	−0.01	<0.01	−2.49^*^	0.99	0.36	0.14	2.61^**^	1.44
Watching gambling online (pre-recorded)	0.37	0.08	4.42^***^	1.45	−0.01	<0.01	−2.77^**^	0.99	0.35	0.14	2.46^*^	1.41

**Notes.**

Behaviours are measured on a frequency scale of 0–3. Gender is coded as 0–1, with 0 being female.

* Predictors that are significant at the *p* < 0.05 level are marked.

** *p* < 0.01 are marked.

*** *p* < 0.001 are marked.

First, the ability of age, gender, and gaming-related behaviours to predict disordered gaming were analysed via a series of logistic regressions. Each regression had disordered gaming (non-disordered, disordered) as predictor. Each analysis had gender, age, and a single form of gaming-related behavior as outcomes. Assumptions of multicollinearity were addressed via the calculation of VIFs for each regression; in each case, all VIFs were below 4, indicating a lack of multicollinearity. Results of logistic regression are depicted below as Table 6.

**Table 6:** Logistic regression of the association between gaming-related behaviours, gender, and age on disordered gaming. Odds Ratios are reported as a measure of effect size.

Gaming-related behaviour					Gender				Age			
Behaviour name	*β*	SE	z	OR	*β*	SE	z	OR	*β*	SE	z	OR
Esports betting	0.31	0.11	2.73^**^	1.36	0.51	0.25	2.08^*^	1.67	−0.04	0.01	−4.64^***^	0.96
Loot box spending	0.69	0.12	5.83^***^	2.00	0.32	0.25	1.27	1.38	−0.03	0.01	−3.51^***^	0.97
Social casino play	0.39	0.12	3.33^***^	1.47	0.50	0.25	2.03^*^	1.65	−0.04	0.01	−4.70^***^	0.96
Real-money video gaming	0.41	0.18	2.23^*^	1.50	0.51	0.24	2.09^*^	1.67	−0.04	0.01	−4.72^***^	0.96
Token wagering	0.66	0.16	4.21^***^	1.94	0.49	0.25	1.96	1.63	−0.04	0.01	−4.23^***^	0.96
Watching loot boxes (live)	0.77	0.14	5.53^***^	2.16	0.29	0.26	1.12	1.33	−0.03	0.01	−3.06^**^	0.97
Watching loot boxes (pre-recorded)	0.64	0.13	5.08^***^	1.90	0.30	0.25	1.19	1.35	−0.03	0.01	−2.88^**^	0.97
Watching gambling online (live)	0.53	0.11	4.98^***^	1.71	0.42	0.25	1.66	1.52	−0.03	0.01	−3.90^***^	0.97
Watching gambling online (pre-recorded)	0.44	0.11	4.04^***^	1.55	0.34	0.25	1.33	1.40	−0.04	0.01	−4.23^***^	0.96

**Notes.**

These represent the influence of a single point difference in a factor on the likelihood that an individual is classified as a disordered gamer. Behaviours are measured on a frequency scale of 0–3. Gender is coded as 0–1, with 0 being female.

* Predictors that are significant at the *p* < 0.05 level are marked.

** *p* < 0.01 are marked.

*** *p* < 0.001 are marked.

It was suggested that the relationship between disordered gaming and problem gambling was calculated. Spearman’s rho for these scores was calculated at 0.26 (*p* < 0.001), indicating a small to moderate relationship between these variables (equivalent *r*^2^ = 0.06).

We then calculated the proportion of individuals who played games with loot boxes that spent money on them. Overall, 283 of the 1,081 individuals in our sample had played a game with a loot box in it (26.1%). Of these 283, the majority (*n* = 160) had spent money on them (56.5%).

Further exploratory analyses were then suggested and conducted on measurements regarding the precedence of gaming-related behaviours and traditional gambling. A subset of individuals in the study indicated that they had both engaged in some form of traditional gambling, and also indicated that they engaged in one of several forms of gaming-related behavior (esports betting, loot box spending, real money gaming, social casino spending, token wagering). These individuals were given a follow-up question for each practice, asking them which one they thought came first.

Finally, one may suggest that individuals may interpret items within the PGSI as referring to gaming-related activities rather than traditional gambling, leading to correlations between engagement in these activities and problem gambling. For example, one question asks individuals whether they have bet more than they could afford in the past 12 months. One might imagine an individual who thinks that they have ‘bet more than they could afford’ on loot boxes, and hence rates this question highly. In order to address whether this might occur, we counted how many individuals within our sample had a rating greater than zero on the PGSI despite not engaging in any traditional form of gambling. This was a rare occurrence: it was only the case for 6 participants.

## Discussion

This study suggests that game-related gambling and gambling-like behaviours may be relatively widespread amongst UK adults. It also provides initial evidence that all of these practices are significantly linked to both problem gambling and disordered gaming.

Overall, a significant proportion of the sample had engaged in some form of gaming-related gambling or gambling-like practice (18.5%). Within our sample, many of these practices appeared as widespread as some traditional forms of gambling. For example, engagement in game-related practices was of comparable popularity to engagement in well-established forms of traditional gambling such as playing slot machines online (14.4%) or betting on horse or dog racing (21.7%). Indeed, just engaging in loot box spending (7.8%) was of similar popularity to engaging in established gambling activities such as playing games of skill for money (6.8%) and playing casino games in a casino (7.4%). However, it is also important to note that whilst the popularity of these specific gaming-related practices may be comparable with engagement in specific forms of gambling, overall engagement with any form of traditional gambling within our sample was much higher: 71.3% of the sample stated that they had engaged in some form of traditional gambling in the past year.

It is interesting to note that this overall summary statistic tallies very closely with the most estimates published in the most recent British Gambling Prevalence Survey (BGPS) (Wardle et al., 2011). In the BGPS, 73% of adults aged 16+ had engaged in gambling in the previous year: here, we see an overall engagement rate of 71.3%. However, it is also key to point out that the rates obtained here also diverge in places from the measurement of prevalence in the BGPS. For example, scratch card use in the BGPS is estimated at 24% of the population. By contrast, 34.7% of participants in our study had purchased scratch cards in the past year. Most strikingly, two percent of participants in the BGPS had engaged in playing online slots in the past year However, 14.4% of our sample stated that they had done this. The reason for this discrepancy is unclear: it seems likely that specific practices may have changed in popularity in the decade separating our sampling from the most recent British Gambling Prevalence Survey. For example, online play may logically have increased in popularity during this period. However, it may be the case that the sample that we obtained overestimates the true national prevalence of specific gambling activities.

Engagement with video-game related gambling practices in general was significantly linked to problem gambling (rho = 0.23). Indeed, every single form of game-related gambling and gambling-like practice was significantly linked to problem gambling, including previously unstudied practices like token wagering and real-money video gaming.

One might assume that the form of video-game related gambling practice that would be most strongly linked to problem gambling was engagement in esports betting, as engagement in esports betting literally constitutes gambling and may therefore directly contribute to problem gambling severity.

However, whilst there was an important link between esports betting and problem gambling, there was an equally strong link between social casino spending and problem gambling (both rho = 0.21). Furthermore, links between problem gambling and watching gambling live on game streaming services like *Twitch* was of comparable magnitude (rho = 0.20). All of these links are of clinically-significant size. The phrase ‘clinically significant’ in this context refers to a benchmark cut-off of *r* = 0.2 that is commonly used in media effects research to indicate an effect that may be of practical importance to clinicians(Ferguson, 2009).

The reason for these links is unclear: it may well be the case, for example, that watching others gamble live might prompt an individual to gamble themselves, and therefore lead to the development of problem gambling. Similarly, one might propose that engagement with simulated games of chance in a social casino context leads to individuals engaging in gambling, and hence the development of gambling problems. However, the direction of any causal link between these activities is unclear and should be the subject for further research. This is of particular importance when it comes to the live viewing of gambling activities on video game streaming services, which has thus far not been explored widely in the literature.

A significant relationship was observed here between the frequency with which an individual engages in loot box spending and their problem gambling. However, interestingly, the relationship seen here was smaller in size than effects observed previously. Previous work has observed a link between the amount of money that an individual spends on loot boxes in a single month and their problem gambling severity of approximately *η*^2^ = 0.05 to 0.12 (Zendle, Meyer & Over, 2019; Zendle & Cairns, 2018). The link between the frequency of engagement in loot box spending and problem gambling observed here was smaller: rho = 0.14, equivalent to *η*^2^ = 0.02. The reason for this difference in magnitude is unclear. It may be the case that previous studies have overestimated the size of this link; it may be that the way that engagement with loot box spending was measured here has conversely led to an underestimation of effect sizes in this instance. Further work is necessary to determine which of these is the case.

A potentially important relationship was observed between disordered gaming and game-related gambling and gambling-like behaviours. Every single form of game-related practice was linked to disordered gaming. Indeed, overall engagement in these behaviours was strongly linked to disordered gaming (rho = 0.43). In other words, over 18% of the variance in gaming-related practices within the sample could be accounted for by the existence of a disordered relationship with video gaming. The specific practices that displayed the strongest links with disordered gaming all related to loot boxes: loot box spending (rho = 0.41), watching loot box openings live online (rho = 0.32), and watching pre-recorded loot box openings (rho = 0.35). This echoes previous research which has established a link between loot boxes and disordered gaming (Li, Mills & Nower, 2019). However, again, the specific reason for this link is unclear, and further qualitative and experimental work is necessary to determine precisely both why disordered gamers might disproportionately engage with loot boxes, and what the consequences of this engagement might be.

The more that individuals engaged in gaming-related gambling and gambling-like practices, the more likely they were to also engage in traditional gambling practices (rho = 0.13). Gaming-related practices were particularly strongly linked to computer-mediated forms of gambling such as online slot machines (rho = 0.27), and online casino games (rho = 0.30). This is logical, considering all these activities necessarily involve using a computer or mobile device.

There were also significant links between all forms of video-game related gambling practices and each other. For example, social casino spending was linked to real money video-gaming (*r* = 0.38), token wagering (rho = 0.34) and esports betting (rho = 0.28). Similarly, loot box spending was strongly to both watching pre-recorded videos of loot box openings (rho = 0.42) and live streams of individuals opening loot boxes (rho = 0.44). These results suggest that individuals may commonly engage in a variety of linked video-game related gambling activities in tandem, rather than a single activity (such as loot box opening or esports betting) in isolation. Indeed, it may well be the case that engagement in specific activities may contribute to engagement in others.

An interesting point may be drawn from these analyses regarding potential third factors that may be responsible for any association between loot box spending and problem gambling. During review, it was suggested that the relationship between loot box engagement and problem gambling may be a product of technological access: individuals who are able to access loot boxes may also be able to access technology such as online betting. Further work is necessary to determine whether this is the case.

Similarly, it is interesting to note that gaming-related behaviors explained only approximately 5% of the variance in problem gambling whereas they explained over 18% of the variance in disordered gaming. One might suggest that this means that the convergence between gaming and gambling is not as important as one might expect. However, we would argue that this is not the case. In terms of magnitude, the observed relationship between problem gambling and gaming-related behaviours is stronger than relationships previously observed between problem gambling and well-known risk factors such as drug abuse and neighborhood disadvantage (Welte et al., 2006). It would be incorrect to state that the magnitude of the observed correlation makes it necessarily unimportant. In fact, we would argue that it is instead the case that the relationship between gaming-related behaviours and disordered gaming is surprisingly large, and bears further study.

## Limitations

There are several potential limitations of this study that must be considered. To begin with, a sceptic may suggest that the sample that was recruited may have self-selected into the study on the basis of interest in the topic under analysis. If this were the case, one might argue that the sample used here is equivalent to a convenience sample. However, this is not the case. As noted in our method section, instructions prior to beginning the study were deliberately neutral, and only 21 participants did not complete the study after beginning it. In a sample of over 1000 participants, this represents a very small threat regarding self-selection bias.

A second point may be raised regarding the generalizability of the data uncovered here: A sceptic may suggest that the recruitment method employed (quota sampling) is unable to achieve a representative sample. Indeed, as early as 1952, the statistician Claus Moser noted that “some experts believe the quota method to be …almost worthless. Others think that …quota sampling can be made highly reliable, and that the heavy extra cost of random sampling does not result in a sufficient increase in accuracy to be worthwhile” (Moser, 1952). This debate has continued through the years (Cumming, 1990; Guignard et al., 2013); T. M. F. (Smith, 1983). Whilst random sampling techniques may therefore represent a researcher’s best chance at reliably maximizing the representativeness of a sample, it is overly reductive to dismiss design based around quota sampling as unable to represent a population under study. Indeed, such designs are commonly employed to do exactly this (e.g., Owen, McNeill & Callum, 1998; Rubin et al., 2005; Swami, 2012).

As stated above, random sampling techniques provide the best chance for researchers to obtain a maximally representative sample. This study employs purposive sampling, and interpretation of prevalence from it may only be done with caution. Furthermore, within the sample itself, the prevalence of disordered gambling observed here appears high (2.3%). Estimates of this magnitude have previously been observed in large-scale samples recruited via random sampling methods (Economou et al., 2019; Effertz et al., 2018; Volberg, Nysse-Carris & Gerstein, 2006). However, in general, the incidence of problem gambling is usually estimated at less than 2% of a national population (Calado & Griffiths, 2016). Further caution must therefore be taken when using the figures reported here as national prevalence estimates. These estimates should be tested via significant subsequent work incorporating large-scale random sampling techniques.

Additionally, common method bias was tested for in this study using an exploratory Harman’s Single Factor Test. However, as noted in (Podsakoff et al., 2003), this method is often overly incautious, and rejects the presence of bias when it really may be present. Whilst all approaches to detect common method bias have limitations, more accurate methods incorporate the measurement of latent methods factors, and the incorporation of these into formal statistical analyses (Podsakoff et al., 2003). Further research should incorporate these statistical methods. It should also focus on the measurement of factors using different and unrelated measures in order to address common method bias issues. More specifically, future research should focus on the measurement and correlation of behavioral and psychometric measures: for example, a correlation between a raw measurement of money spent on loot boxes in a video game with an individual’s problem gambling severity.

An additional limitation of this study may be raised regarding the framing of our questions: as noted in our method section we endeavored to make our items as human-readable and interpretable as possible. However, it is credible that some participants may not have understood some of the questions that they were responding to. Further qualitative and scale development work is necessary to address these issues.

The most important limitation of this research is, however, rooted in its well-explored status as a cross-sectional study: whilst it may be able to uncover and measure correlations between factors, it cannot establish precedence. Whilst gaming-related behaviours are linked to both problem gambling and disordered gaming, it is not clear which preceded or caused the other. For example, loot box spending may be linked to disordered gaming because the gambling-like mechanisms present in loot boxes entrap gamers, leading to the development of disordered gaming. However, it is just as credible that these factors share a relationship because individuals with gaming disorder tend to play games more frequently and for longer periods of time, and hence are more likely to buy loot boxes. Further longitudinal studies are needed to establish Grainger causality. Significant experimental work is needed to establish the existence of other forms of causality.

## Conclusions

There is one clear conclusion that may be drawn from these results: the convergence of gaming and gambling is far more complex than simply the existence of loot boxes. There are a broad spectrum of inter-connected video game practices that are also similar to gambling. Many of these activities appear widespread amongst UK adults.

The widespread nature of these practices is important because all of them are significantly linked to both problem gambling and disordered gaming. In many cases these links are of a clinically significant magnitude.

The causal nature of these links is unclear. It may well be the case that engagement in practices like real-money video gaming and social casino spending are linked to problem gambling because individuals with pre-existing gambling problems are more likely to engage in these activities. However, it may also be the case that these links represent a situation in which the existence of a diverse ecosystem of gambling-like activities in video games is driving the creation of problem gambling amongst video gamers. Given the widespread nature of these practices that is suggested here, this may pose an important public health risk. Significant further work is urgently needed to further investigate these phenomena.

## Additional Information and Declarations

### Data Availability

The following information was supplied regarding data availability: All datasets that are relevant to this study are available at OSF: Zendle, D. (2019). “Gambling Gaming Prevalence”. OSF. Dataset. https://osf.io/3jpey/.
